# Widespread and progressive brain atrophy is a common feature in patients with mitochondrial disease

**DOI:** 10.1007/s00415-025-13354-z

**Published:** 2025-09-23

**Authors:** Nora Mickelsson, Jussi Hirvonen, Mika H. Martikainen

**Affiliations:** 1 Clinical Neurosciences, Department of Clinical Medicine, University of Turku, Turku, Finland; 2 Neurocenter, Turku University Hospital, Turku, Finland; 3 Department of Radiology, Turku University Hospital, Turku, Finland; 4 Department of Radiology, Faculty of Medicine and Health Technology, University of Tampere, Tampere, Finland; 5 Research Unit of Clinical Medicine, Neurology, University of Oulu, Oulu, Finland; 6 Neurocenter and Medical Research Center, Oulu University Hospital, Oulu, Finland; 7 Present Address: Faculty of Medicine, University of Oulu, Aapistie 5A, 90220 Oulu, Finland

**Keywords:** Mitochondrial disease, Cerebral atrophy, Magnetic resonance imaging, Stroke-like episodes, Neurodegeneration, Longitudinal imaging

## Abstract

**Background:**

Primary mitochondrial diseases comprise a group of inherited disorders that frequently affect the central nervous system. Previous studies have reported brain imaging findings commonly associated with mitochondrial disease. However, longitudinal data on volumetric brain abnormalities, their progression in time, and associations with clinical features of the disease remain limited.

**Methods:**

We conducted a retrospective observational study of 36 patients with genetically confirmed mitochondrial disease at Turku University Hospital (Turku, Finland). A total of 73 brain magnetic resonance scans (1–8 per patient) were analysed using the cNeuro® image quantification tool to assess lobar and regional cortical atrophy. Associations with clinical features, including stroke-like episodes (SLEs), sex, and genetic subtype, were investigated.

**Results:**

Cerebral atrophy was present in all patients and was most pronounced in the temporal and occipital lobes. Patients with a history of SLEs exhibited significantly greater atrophy in both temporal lobes and the right occipital and parietal lobes. Follow-up imaging (available for 15 patients) revealed progressive atrophy, particularly in the occipital lobes, in patients with SLEs. No significant differences in atrophy severity or progression were found between patients with the m.3243A > G variant and those with other genetic causes.

**Conclusions:**

Cerebral atrophy is a common and often progressive feature of mitochondrial disease, even in patients without clinical brain symptoms. Atrophy predominantly affects posterior brain regions, and its progression is particularly evident in patients with SLEs. These findings underline the neurodegenerative nature of mitochondrial disease and highlight the need to develop neuroprotective therapies.

## Introduction

Primary mitochondrial diseases are inherited neurometabolic disorders caused by pathogenic variants in either mitochondrial DNA (mtDNA) or nuclear DNA (nDNA), affecting mitochondrial oxidative phosphorylation, the core process of cellular energy production. To date, over 350 disease-causing genes have been identified [1]. The estimated prevalence of mitochondrial disease is 1:4300 [2]. Recently, the prevalence of mtDNA disease in the adult population of Southwestern Finland was estimated at 9.2/100000, and disease related to the common m.3243A > G variant at 4.2/100000 [3].

Mitochondrial diseases frequently affect the central nervous system (CNS), particularly the brain [4, 5]. A characteristic manifestation is the stroke-like episode (SLE), a subacute evolving neurological syndrome [6]. Brain atrophy, particularly in the cerebellum, has been reported as a common imaging finding in patients with mitochondrial disease. Other reported abnormalities in brain imaging include white matter changes, basal ganglia calcifications, and focal lesions, e.g. stroke-like lesions (SLLs) and lacunar infarcts [7–10].

The most common pathogenic mtDNA variant is m.3243A > G in the mitochondrial leucine (UUR) tRNA gene [2, 3, 11], originally described in patients with the mitochondrial encephalomyopathy, lactic acidosis, and stroke-like episodes (MELAS) syndrome. Patients harbouring the m.3243A > G variant exhibit both grey and white matter volume reduction, with or without SLEs. Grey matter volume reduction is greater in patients with SLEs [7, 9]. In patients with m.3243A > G variant higher mutation loads, and cerebral symptoms have been associated with more severe brain atrophy [12–14]. Atrophy is reported being most pronounced in the occipital lobes and the cerebellum [7], although some studies have identified more widespread atrophy involving the frontal, parietal, and temporal lobes [9, 13]. SLLs observed during SLEs are known to predominantly affect the posterior regions of the brain, but over time they may evolve, spread to adjacent areas, and result in volume loss due to cortical laminar necrosis [10, 15, 16].

Although the understanding of mitochondrial disorders in terms of the spectrum of clinical features as well as the genetic diagnosis has advanced significantly in recent years, knowledge about the extent, distribution, and progression of brain atrophy in mitochondrial disease still remains quite limited, particularly across different genetic aetiologies and clinical phenotypes. This information is of particular importance given the frequent neurological involvement and progressive nature of mitochondrial diseases [17–19]. We utilised the cNeuro® MR image tool to quantitatively assess brain volumes as well as the distribution and progression of brain atrophy in a well-characterised single centre cohort of mostly adult patients with mitochondrial disease, focusing on associations with genetic aetiology, clinical phenotype, and sex.

## Methods

This retrospective, observational study was based on the mitochondrial disease patient cohort at Turku University Hospital (TUH; Turku, Finland) in the region of Southwest Finland. Most of these patients with mitochondrial disease had regular follow-ups at TUH. Patient data and brain MR images were obtained from the TUH electronic medical record system (earliest available date January 1, 2000). The data collection was completed in 2023. This research was covered by the TUH research permission TO4/016/16. Individual informed consent was not required for this retrospective, register-based study.

Several different MR imaging scanner models (1.5 T and 3 T) were used from the year 2000 onwards. The imaging protocol included a three-dimensional T1-weighted gradient-echo sequence (3D T1-w) and a fast fluid-attenuated inversion recovery (FLAIR) sequence. MR images were analysed using the cNeuro® MR image quantification tool [20] to evaluate global cortical atrophy (GCA) [21] in five different brain regions: cerebral cortex, frontal lobe, temporal lobe, parietal lobe, and occipital lobe, in both hemispheres. GCA reflects the degree of cortical atrophy on a four-point scale (0–3), where 0 indicates no atrophy and 3 severe atrophy based on comparisons with brain region volumes in a reference population. This method enables the evaluation of the severity of cortical atrophy at individual patient level without the need for control groups and is validated for use in both clinical and research settings in neurodegenerative diseases [22]. The cNeuro cMRI has clearance of the United States Food and Drug Administration and is also in clinical use at TUH.

GCA scores were normalized for age, sex, and head size. GCA data were non-normally distributed, as indicated by the Shapiro–Wilk test (all p < 0.05). Therefore, non-parametric statistical models were used. First, GCA data were compared to zero median to investigate atrophy across all participants using the one-sample Wilcoxon signed-rank test. Second, group differences in GCA were tested using the Mann–Whitney *U* test. Finally, atrophy progression was assessed individually for each patient with multiple available brain MR images by subtracting the GCA scores between the first and last MR imaging and tested using the Wilcoxon signed-rank test. The change in GCA score between the first and last image was divided by the duration of the follow-up period to obtain an annualised rate of progression. Statistical analyses were performed with IBM SPSS Statistics for Windows version 29. Statistical significance was set at *p* value < 0.05.

## Results

Seventy-three brain MR imagings from 36 patients (15 men, 21 women) were analysed. 15 patients (42%) had more than one brain imaging available (range 2–8 images/patient). The mean interval between patients’ first and last brain MR imaging was six years (range 0.5–15 years). The most common molecular diagnosis was the m.3243A > G variant in mtDNA (N = 16) (Table 1). Mean age at earliest available brain imaging was 43 years (range 0.6–75 years). Twelve patients had a history of at least one SLE; these patients have been reported in detail elsewhere [23]. Fifteen patients had a history of some brain symptoms, including ataxia, encephalopathy, epilepsy, and SLEs.

**Table 1:** Demographic and clinical features of the investigated patients with mitochondrial disease

	N (%)	Sex (F/M), N (%)	Current age or age at death, mean, years (range)	History of SLE, N (%)	Presence of brain symptoms, N (%)	Age at first brain MRI, years (range)	Brain MRIs/patient, mean (range)
All	36	F 21 (58) M 15 (42)	51 (2–84)	12 (33)	15 (42)	43 (0.6–82)	2.0 (1–8)
M.3243A > G	16 (44)	F 9 (56) M 7 (44)	49 (11–79)	7 (44)	7 (44)	42 (5–66)	2.1 (1–5)
POLG^a^	2 (6)	F 2 (100)	25 (20–30)	2 (100)	2 (100)	20 (17–22)	5.0 (2–8)
Other mDNA mutations^b^	15 (42)	F 8 (53) M 7 (47)	59 (2–84)	3 (20)	6 (40)	52 (0.6–82)	1.8 (1–4)
Other nDNA mutations^c^	3 (8)	F 2 (67) M 1 (33)	33 (29–40)	0	0	19 (10–30)	1

F, female; M, male; MRI, magnetic resonance imaging; mtDNA, mitochondrial DNA; nDNA, nuclear DNA; SLE, stroke like episode

^a^W748S homozygous variant in *POLG*

^b^m.11778G > A *N* = 6, m.8344A > G *N* = 2, m.8993 T > G, m.3271 T > C, m.13513G > A, m.10427G > T, m.3260A > G, mtDNA single deletion, axial myopathy with multiple mtDNA deletions, no definitive molecular genetic diagnosis (*N* = 1 in all)

^c^compound heterozygous mutations c.228-20_21delTTinsC (p.R76SfxX5) and c.492 + 2 T > C (p.M134_K185del) in the DARS2 gene (*N* = 3)

**Fig. 1:**
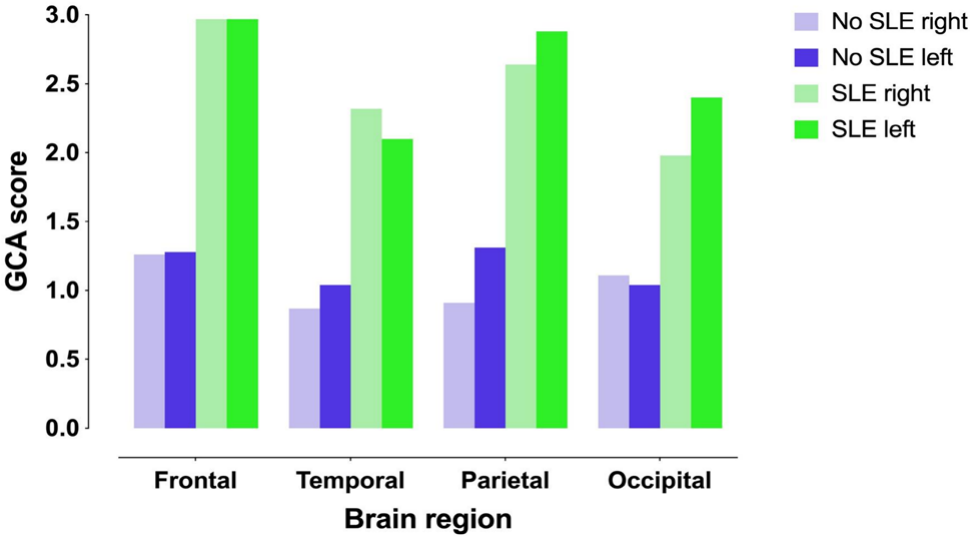
Global cortical atrophy scores indicating widespread brain atrophy across different brain regions among patients with mitochondrial disease. GCA, Global cortical atrophy; SLE, Stroke like episode

In the earliest brain MR imagings, atrophy was statistically significant in all brain regions (*p* < 0.001), being most pronounced in the temporal lobe (mean GCA 1.33 on the right and 1.36 on the left) and in the right occipital lobe (1.34). There was more atrophy in the temporal, occipital, and right parietal lobes in women (*p* < 0.05). Patients with a history of SLE had more brain atrophy in both temporal lobes and in the right occipital and parietal lobe than those without a history of SLE (*p* < 0.05) (Table 2 and Fig. 1). No statistically significant differences in brain atrophy in different regions were found when comparing patients with the m.3243 A > G variant to those with other genetic causes.

**Table 2:** Cerebral atrophy in patients with or without a history of stroke like episodes

	SLE (*N* = 12)	No SLE (*N* = 24)	
GCA (0–3)	median, IQR	median, IQR	*P* value^a^
Frontal lobe, right	2.97 (2.01)	1.26 (0.41)	0.109
Frontal lobe, left	2.97 (2.17)	1.28 (0.55)	0.125
Temporal lobe, right	2.32 (2.44)	0.87 (1.01)	0.03
Temporal lobe, left	2.10 (2.48)	1.04 (0.86)	0.026
Parietal lobe, right	2.64 (2.00)	0.91 (0.74)	0.038
Parietal lobe, left	2.88 (2.04)	1.31 (0.62)	0.080
Occipital lobe, right	1.98 (2.04)	1.11 (1.11)	0.034
Occipital lobe, left	2.40 (1.78)	1.04 (0.91)	0.103

GCA, global cerebral atrophy; IQR, Interquartile range; SLE, stroke like episode.

^a^Mann–Whitney U-test

**Fig. 2:**
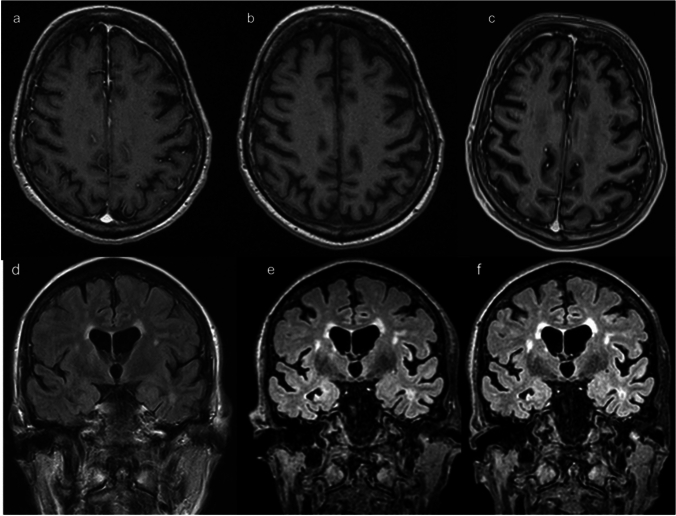
Progressive brain atrophy associated with mitochondrial disease. **a-c** Patient with the m.3243A > G variant. a. T1 + contrast, patient age 61y; b. T1, 64 y; c. T1, 66y. **d-f** Patient with axial myopathy with multiple mtDNA deletions, no definitive molecular genetic diagnosis. d. flair, 76y; e. flair, 79y; f. flair, 81y

Brain atrophy progression was greatest in the left temporal lobe and the right parietal lobe (mean GCA change: 0.062/year for the temporal and 0.005/year for the parietal lobe) but did not reach statistical significance in the whole sample (*N* = 15). Atrophy progression rate in the right parietal lobe was greater in men (*p* < 0.05). Patients with a history of SLE (*N* = 10) had greater atrophy progression in the left (*p* = 0.003) and in the right (*p* = 0.040) occipital lobes compared to those without (*N* = 5). There was no significant difference in atrophy progression in patients with the m.3243A > G variant (*N* = 7) compared to others. Figure 2 demonstrates the observed progressive brain atrophy in two patients.

## Discussion

We used the cNeuro® MR image tool to quantitatively evaluate global cortical atrophy in 36 mostly adult patients with genetically confirmed mitochondrial disease related to various mitochondrial and nuclear DNA pathogenic variants, the m.3243A > G in mtDNA being the most common aetiology. Significantly lower brain volumes compared to those in age and sex adjusted reference population were observed in all patients, independent of the presence of brain symptoms and the underlying genetic variant. Brain atrophy was most prominent in the temporal lobes and in the right occipital lobe. Sensorineural hearing loss is common in mitochondrial disease, and while typically cochlear in origin [24], cortical involvement, particularly after SLEs, has also been reported [25]. Given the location of the auditory cortex in the superior temporal gyrus, the pronounced temporal atrophy in our cohort may suggest cortical auditory involvement, although this cannot be confirmed with the lobar-level resolution used.

The absence of significant differences in brain atrophy in all regions between patients with specific genetic variants suggests a uniform pattern of atrophy related to mitochondrial dysfunction, independent of the underlying genetic variant. The presence of brain atrophy also in patients lacking brain symptoms suggests that neurodegeneration is a common characteristic in mitochondrial diseases. In line with this finding, a previous study revealed a global decrease in brain oxygen consumption in patients with mitochondrial disease and the m.3243A > G variant, independent of cerebral symptoms [12].

Patients with a history of SLEs exhibited greater atrophy in both temporal lobes, as well as in the right occipital and parietal lobes, compared to those without a history of SLE. Previous studies have also reported an association between brain symptoms and increased atrophy [12], and volume reduction extends beyond the regions previously affected by stroke-like lesions (SLL) [9]. Consistent with these findings, in our recent study we also found that SLLs were primarily located in the parieto-occipital regions [23]. Increasing evidence indicates that the pathogenesis of SLEs is primarily driven by metabolic energy failure caused by respiratory chain dysfunction, leading to neuronal hyperexcitability and secondary neurovascular changes that mimic, but are distinct from, classical ischemia [26–28]. Histopathological studies in patients with SLEs have also shown high mutated mtDNA loads and associated COX deficiency in leptomeningeal and cortical vessels, particularly in the posterior cortex [29], suggesting mitochondrial dysfunction of brain vasculature may also contribute. Therefore, the more pronounced atrophy observed in patients with SLEs is likely driven primarily by metabolic dysfunction, with vascular mitochondrial impairment as an additional factor.

There are few studies on the progression of brain atrophy in patients with mitochondrial disease. While progression of clinical manifestations in patients with the m.3243A > G variant has been demonstrated, a previous study reported atrophy progression on brain MRI in only three of 20 patients [8]. In another earlier study of patients with MELAS (all of whom were children with a history of SLEs) generalized brain atrophy progressed in six of the seven cases, with the most pronounced changes in the posterior cerebral hemispheres [30]. Progressive cerebral atrophy has also been reported in 4 out of 17 MELAS patients with clinical onset in early adulthood [10]. In patients with POLG variants, progressive brain volume loss has been reported in the occipital and thalamic regions [31], as well as in the right pallidum [32].

In the present study, the 15 patients with available follow-up imaging (2–8 images), the greatest progression of brain atrophy was observed in the left temporal lobe and in the right parietal lobe. Atrophy progression in the occipital lobes was more pronounced in patients with a history of SLEs compared to those without. This finding is likely explained by the predominance of SLLs in posterior brain regions, the known spatial progression of these lesions [16] and the greater number of available follow-up imaging studies in patients with multiple SLEs.

The m.3243A > G mutation, common in our cohort, has been shown to impair mitochondrial protein synthesis leading to respiratory chain dysfunction and energy failure, particularly affecting complexes I and IV [33–35]. This leads to neuronal energy failure, offering a plausible cause for the progressive cortical atrophy observed in metabolically active brain regions. In the present study, faster progression of brain atrophy in the right parietal lobe was observed among men. This sex difference could be random and related to the relatively small sample size. However, a real sex difference is also possible and could be related to the *“mother’s curse”*, referring to maternally inherited mtDNA variants that preserve specifically male-deleterious effects [36, 37], or to hormonal influences, with oestrogen supporting mitochondrial function and reducing oxidative stress, potentially providing females with greater neuroprotection [38].

A key strength of this study is the use of a quantitative and validated imaging analysis tool (cNeuro®) in a clinically and genetically well-characterized cohort of patients with mitochondrial disease. Individual data on the clinical phenotype and brain imaging were collected over an extended period, enhancing the longitudinal relevance of the findings. The use of the GCA scale, which is inherently adjusted for demographic variables such as age and sex, further supports the applicability of our results at the individual patient level. The absence of a control group does not limit the internal comparison between patients with and without a history of SLEs.

The study has certain limitations. The retrospective design introduces variability in imaging protocols and follow-up intervals. In addition, the relatively small cohort size and heterogeneity of genetic diagnoses limits statistical power, particularly in subgroup analyses. Furthermore, the potential correlation between disease duration and the severity of brain atrophy could not be assessed with confidence, as precise determination of disease duration was not possible for all patients. In addition to cortical atrophy, other imaging abnormalities such as white matter changes and stroke-like lesions were observed in brain images. In the present study, these were not analysed in detail.

Overall, the results of the present study reveal that brain atrophy is a common and progressive feature in patients with mitochondrial disease, regardless of genetic subtype or the presence of clinical brain symptoms such as ataxia, encephalopathy, epilepsy, or SLEs. Brain atrophy progression was particularly evident in patients with SLEs and most pronounced in the temporal and occipital lobes, reflecting the special vulnerability of these brain regions. These findings underline the neurodegenerative nature of mitochondrial disease and highlight the need to develop neuroprotective therapies.

## Data Availability

Individual level patient data is not available. Anonymised aggregate data is available from the corresponding author on reasonable request.
